# Pharmacoeconomic analysis (CER) of Dulaglutide and Liraglutide in the treatment of patients with type 2 diabetes

**DOI:** 10.3389/fendo.2023.1054946

**Published:** 2023-01-23

**Authors:** Yu Su, Shuo Zhang, Zezhen Wu, Weiting Liu, Jingxian Chen, Feiying Deng, Fengwu Chen, Dan Zhu, Kaijian Hou

**Affiliations:** ^1^ Center of Teaching Evaluation and Faculty Development, Anhui University of Chinese medicine, Hefei, Anhui, China; ^2^ Medical College of Shantou University, Shantou, China; ^3^ Department of Endocrine and Metabolic Diseases, The First Affiliated Hospital of Shantou University Medical College, Shantou, China; ^4^ Department of Endocrine and Metabolic Diseases, Longhu People’s Hospital, Shantou, China; ^5^ School of nursing, Anhui University of Chinese medicine, Hefei, Anhui, China; ^6^ School of Public Health, Shantou University, Shantou, China

**Keywords:** Dulaglutide, type 2 diabetes, treatment effect, pharmacoeconomics, insulin resistance index, cost-effectiveness ratio

## Abstract

**Aim:**

To evaluate the treatment effect Fand pharmacoeconomic value of Dugaglutide in women with type 2 diabetes.

**Methods:**

Women (n=96) with type 2 diabetes recruited from June 2019 to December 2021 were randomized into two equal groups. The control group was treated with Liraglutide, and the observation group was treated with Dulaglutide, both for 24 weeks. The blood glucose levels, biochemical index, insulin resistance index (HOMA-IR), cost-effect ratio (CER), and drug safety were determined and compared between the two groups.

**Results:**

Blood glucose levels, the biochemical index, and HOMA-IR were lower in both groups after the treatment (*P* < 0.05), and there was no statistical difference in the blood glucose levels, biochemical index and HOMA-IR between the two groups (*P* > 0.05). The CER levels did not differ statistically between the two groups (*P* > 0.05). Both the cost and the incidence of drug side effects during solution injection were lower in the observation group than in the control group after 24 weeks of treatment (*P* < 0.05).

**Conclusion:**

Both Dulaglutide and Liraglutide can reduce blood glucose levels, improve biochemical index, and HOMA-IR levels in women with type 2 diabetes. Dulaglutide is more cost-effective and safe.

**Clinical trial registration:**

https://www.chictr.org.cn/index.aspx, identifier ChiCTR1900026514.

## 1 Introduction

Type 2 diabetes tends to occur in adults because of a continuous increase in the blood glucose level, which is caused by insufficient insulin secretion or difficulty in the use of insulin for various reasons. Among all of the type 2 diabetes patients, evidence suggests that women experience a higher excess mortality than men (1, 2). Persistent hyperglycemia can cause pathological changes in the macrovascular, microvascular, and nervous systems and, in severe cases, damage to the heart and kidney (3). The pathology of type 2 diabetes is complex, and clinical symptoms can include polyphagia, polyuria, polydipsia, and weight loss (4, 5).

In the early 1980s, glucagon-like peptide 1 (GLP-1) was found to be the glucagon-stimulating enzyme cleavage product (6)produced in intestinal L cells. GLP-1, as an intestinal peptide mainly secreted after ingestion of glucose or mixed diet, increases glucose-stimulated insulin secretion at physiological plasma concentration, meeting all standards of incretin hormone (7, 8). The insulin-promoting effect of GLP-1 in type 2 diabetes patients shows that it has a potential role in drug treatment of the disease (9, 10). The most obvious physiological effect of GLP-1 is its insulin-promoting effect (6). It is worth noting that GLP-1 only increases insulin release in the case of hyperglycemia, so it will not lead to hypoglycemia. In addition, GLP-1 inhibits the pancreas α The cells release glucagon, which may be through the islets δ Somatostatin is locally released from the cells to mediate the release of (10, 11). In addition, GLP-1 has many other functions: the central nervous system (CNS) induces satiety and satiety (12), reduces blood pressure (13), and reduces postprandial triglyceride and free fatty acid concentrations. Lilalutide is a GLP-1 receptor agonist. The standard therapeutic dose of liraglutide is 1.2mg once a day. However, if the patient has insufficient blood glucose response to the drug, it is recommended to titrate to 1.8mg once a day. In phase III clinical trial of liraglutide in patients with type 2 diabetes, HbA1c levels were reduced by 1.1 – 1.8% (14, 15).

Liraglutide is a commonly used glucagon-like peptide 1 (GLP-1) receptor agonist. *In vivo*, Liraglutide can bind to GLP-1 receptors on pancreatic beta cells and then stimulate the synthesis and secretion of insulin, which can increase insulin sensitivity in peripheral tissues, enhance insulin-mediated glucose utilization, inhibit hepatic glycogen callogenesis, reduce glucose uptake by intestinal cells and decrease hepatic glucose output (16). Liraglutide also increases satiety by acting on the central nervous system (17) and slowing gastric emptying time, which reduces the total energy intake (18, 19).

Unlike short-acting compounds, long-acting GLP-1 receptor agonists do not appear to substantially affect gastric motility when taken for a long time. Long-acting GLP-1 receptor agonists lack influence on gastric emptying rate76. Dulaglutide is a GLP-1 peptide fused with IgG. Compared with natural GLP-1, Dulaglutide shows extended biological activity due to its extended half-life (~90 hours), which supports the weekly administration of the drug (15). A weekly dose of 0.05 – 8.0mg resulted in a decrease of 0.2 – 1.2% in HbA1c levels after 5 weeks. Compared with short-acting drugs that require more frequent administration, the convenience of injecting long-acting compounds once a day or once a week is an obvious advantage. Patients with frequent changes in daily activities, such as business travelers and shift workers, may prefer long-acting compounds, which can improve patient compliance (20).

**Figure 1:**
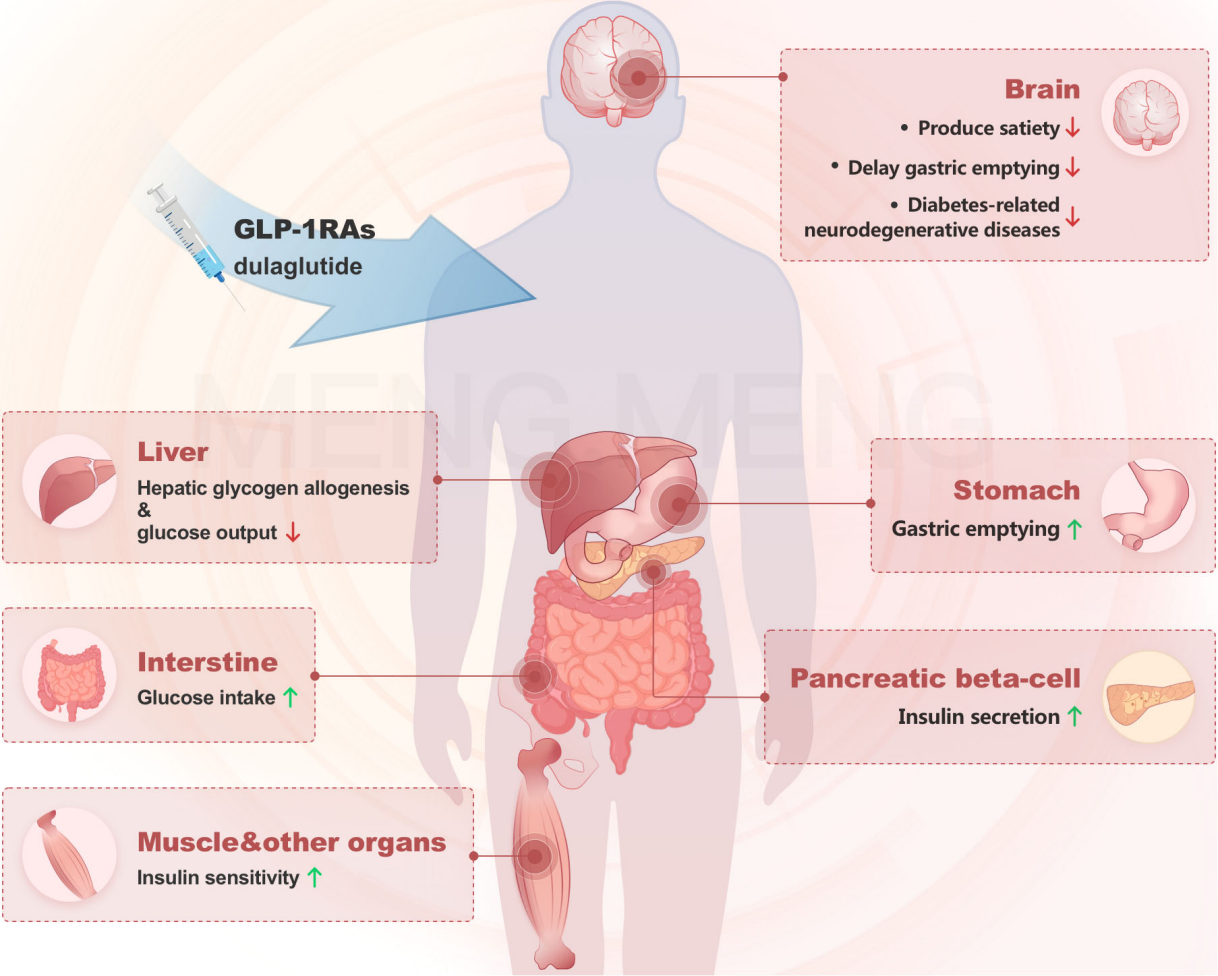
The mechanism of action of Dulaglutide in decreasing blood glucose.

Dulaglutide is one GLP-1 Fc fusion protein that activates GLP-1 receptors and promotes glucose-dependent insulin secretion, which helps reduce fasting and postprandial glucose levels (21). Dulaglutide improves the insulin secretion index and helps regulate the body’s blood glucose level (22). Growing evidence suggests that Dulaglutide has the potential to treat diabetes-related neurodegenerative diseases (23, 24). The mechanism of action for how the drug decreases blood glucose is shown in (**Figure 1**). However, few clinical studies have focused on the economic value of Dulaglutide injection in type 2 diabetic patients (25). This study was conducted to investigate the therapeutic effect and pharmacoeconomic value of Dulaglutide in female patients with type 2 diabetes.

## 2 Materials and methods

### 2.1 Patient recruitment

Female patients (n=96) with type 2 diabetes were recruited from June 2019 to December 2021, ranging in age from 23 to 69 years, and the average age of patients was 46.14 ± 5.78 years (mean ± S.D.); the mean body mass index (BMI) was 22.62 ± 3.71 kg/m^2^ (the BMI levels ranged from18.31 to 29.34 kg/m^2^); and the disease duration was an average 5.73 ± 0.92 years (the range was 1-12 years). We randomly assigned participants at a ratio of 1:1. Using an interactive voice response system, all patients were randomly divided into two groups according to a computer-generated random sequence.

### 2.2 Inclusion and exclusion criteria

The study design included the following inclusion criteria (26): (1) The type 2 diabetes patients were diagnosed based on the American Diabetes Association criteria which specify that the FPG ≥ 126 mg/dL (7.0 mmol/L), 2-h PG ≥ 200 mg/dL (11.1 mmol/L) during OGTT (Oral Glucose Tolerance Test), an A1C level ≥ 6.5% (48 mmol/mol), or in a patient with classic symptoms of hyperglycemia or hyperglycemic crisis, a random plasma glucose ≥ 200 mg/dL (11.1 mmol/L). Fasting is defined as no caloric intake for at least 8 h. The fasting glucose test should be performed as described by WHO, using a glucose load containing the equivalent of 75 g anhydrous glucose dissolved in water, and the test should be performed in a laboratory using a method that is NGSP (National Glycohemoglobin Standardization Program) certified and standardized to the DCCT (Diabetes Control and Complications Trial) assay.(2) No history of allergy and contraindication to Dulaglutide and Liraglutide, and be able to tolerate the treatment. Exclusion criteria included: (1) Patients with other types of diabetes mellitus (DM) rather than T2DM; (2) Patients who have used weight reduction drugs within 24 weeks; (3) Patients with clinically significant hepatobiliary, renal, cardiovascular, gastrointestinal or autoimmune system disease; (4) Coagulation disorders; (5) Patients who are judged by the investigator as unlikely to comply with the protocol, or patients with serious physical or psychological illnesses that could affect the effectiveness or safety of the study.

### 2.3 Methods

Both groups of patients were admitted to the hospital and underwent stringent blood glucose monitoring. All patients did not use other hypoglycemic drugs. During the study period, the patients’ exercise intensity was medium to low, and they followed the diabetes diet. The control group was injected subcutaneously with Liraglutide (Novo Nordisk Pharmaceutical Co., Ltd., China; at the specification 3 mL: 18 mg/stick) at the following regimen: 0.6 mg once a day during the first week before breakfast; then 1.2 mg once a day, from the 2^nd^ to 24^th^ week. The observation group was treated with Dulaglutide. Patients were given a subcutaneous injection of Dulaglutide every week. The dose of Dulaglutide injected was 0.75mg in the first week, if the blood glucose is not well controlled, the dose can be increased according to the patient’s actual situation, where the range of injection was generally 0.75-1.5 mg for 24 weeks (1 course of treatment).

### 2.4 Endpoints

(1) Glucose metabolism indices. Fasting postprandial glucose(FPG) and 2-hour postprandial glucose(2HPG) levels were measured using a glucose meter before treatment and after 24 weeks of treatment in both groups. Patients’ glycosylated hemoglobin (HbAlc) levels were measured using an automatic biochemical analyzer (27).

(2) Biochemical index and insulin resistance index (Homa-IR). The levels of visceral adiponectin were measured by enzyme-linked immunosorbent assay (2). Leptin (Lp) levels were measured by radioimmunoassay. Fasting-insulin (FINS) was measured by a fully automated immunoluminescence analyzer and HOMA-IR (HOMA-IR=FPG*FINS∕22.5) levels were calculated (17).

(3) Cost-Effectiveness Ratio (CER). The economic value analysis contained two aspects: cost determination and efficacy analysis. A cost-effectiveness ratio (CER) was performed, where the lower CER indicates the better economic value (28). Safety was assessed based on adverse events: the incidence of nausea and vomiting, hypoglycemia, cholecystitis, allergic reactions, and liver and kidney abnormalities (29).

### 2.5 Statistical analysis

Unpaired Student’s t-test for categorical variables was applied for comparison between observation and controlgroups. Results with a two-tailed p-value of <0.05 were considered significant (19 31). IBM SPSS Statistics 25 was used for thedata analyses.

## 3 Results

### 3.1 Changes in glucose metabolism indices

There was no statistical difference in glucose metabolism indices between the two groups before treatment (*P*> 0.05), though FPG, 2hPG, and HbAlc levels were lower in both groups after 24 weeks of treatment (*P*< 0.05). No statistical difference was found in glucose metabolism indices between the observation and control groups after 24 weeks of treatment (*P*> 0.05) (**Table 1**).

**Table 1:** Comparison of glucose metabolism indices between control and observation groups.

Group	n	FPG (mmol/L)		2hPG (mmol/L)		HbAlc(%)	
		W0	W24	W0	W24	W0	W24
Observation group	48	10.59 ± 2.31	6.23 ± 0.89^#^	13.21 ± 2.96	7.12 ± 1.42^#^	7.89 ± 0.93	5.67 ± 0.71^#^
Control group	48	10.62 ± 2.33	8.59 ± 1.52^#^	13.23 ± 2.99	10.97 ± 1.98^#^	7.92 ± 0.96	6.74 ± 0.82^#^
p value	/	0.950	0.000	0.974	0.0000	0.877	0.0000

W0, week 0, before the solution injection treatment; W24, week 24, after the solution injection treatment for the period of 24 weeks; #: P< 0.05 compared with before the solution injection treatment. Data are displayed as mean ± SD.

### 3.2 Comparison of the biochemical index and Homa-IR index between control and observation groups

There was no statistical difference in the biochemical indices and HOMA-IR index between the two groups before treatment (*P*> 0.05). However, the levels of visceral adiponectin, Lipoprotein (LP), FINS. and HOMA-IR were lower than those before treatment in both groups after 24 weeks of treatment (*P*< 0.05). We found no statistical difference in biochemical indices and HOMA-IR levels between the observation and control groups after 24 weeks of treatment (*P*> 0.05) (**Table 2**).

**Table 2:** Comparison of biochemical indices and Homa-IR index between control and observation groups .

Group		Visceral adiponectin (ng/mL)	Lp (μg/L)	FINS (IU/L)	HOMA-IR
Observation group (n=48)	W0	56.49 ± 5.69	5.97 ± 0.92	13.16 ± 1.41	4.34 ± 0.79
	W24	39.45 ± 4.31^*^	3.11 ± 0.49^*^	9.34 ± 0.67^*^	2.21 ± 0.42^*^
Control group (n=48)	W0	56.51 ± 5.72	5.99 ± 0.94	13.18 ± 1.43	4.36 ± 0.81
	W24	40.11 ± 4.34^*^	3.13 ± 0.51^*^	11.32 ± 0.98^*^	2.23 ± 0.44^*^

W0, week 0, before the solution injection treatment; W24, week 24, after the solution injection treatment for the period of 24 weeks; #: P< 0.05 compared with the other group; *: P < 0.05 compared with before the solution injection treatment. Data are displayed as mean ± SD.

### 3.3 Comparison of the Cost and CER between control and observation groups

Both groups completed the continuous treatment over 24 weeks and the clinical application value of the different drugs was assessed from an economic point of view. No statistical difference in CER levels was found between the two groups (*P*> 0.05); however, the cost was lower in the observation group than in the control group after 24 weeks of treatment (*P*< 0.05) (**Table 3**).

**Table 3:** Comparison of the cost and CER between control and observation groups.

Group	n	Cost (RMB)	CER
Observation group	48	7515.69 ± 86.49	48.57 ± 4.31
Control group	48	24596.68 ± 453.69	48.91 ± 4.37
p value	/	0.000	0.702

Data are displayed as mean ± SD.

### 3.4 Comparison of safety between the two groups

The incidence of nausea and vomiting, hypoglycemia, cholecystitis, allergic reactions, and liver and kidney abnormalities was much lower in observation group compared to the control group (**Table 4**).

**Table 4:** Comparison of safety between control and observation groups [n(%)].

Group	n	Nausea and vomiting	Hypo- glycemia	Cholecy- stitis	Allergic reactions	Liver and kidney abnormalities	Total sum
Observ-ation group	48	0(0.00)	1(2.08)	0(0.00)	0(0.00)	0(0.00)	1(2.08)
Control group	48	2(4.17)	2(4.17)	2(4.17)	0(0.00)	1(2.08)	7(14.58);
p value	/	/	/	/	/	/	0.027

Data are displayed as number and percentage(%).

## 4 Discussion

Type 2 diabetic patients account for more than 90% of all diabetic patients. Many patients with type 2 diabetes might not have a complete loss of insulin secretion, and some might have excessive insulin secretion (30). However, type 2 diabetic patients are poor users of insulin, and the persistent hyperglycemic condition will have a negative impact on the ability of the body to metabolize glucose, leading to chronic elevation of blood glucose in patients. Liraglutide is one of the human glucagon plasmin-1 analogues, which belongs to a family of glucose-lowering drugs with a strong hypoglycemic effect. Liraglutide is an injection solution but not insulin, and it can promote insulin secretion and inhibits the secretion of hyperglycemic hormone and the feeding center in the brain.

In our study, both short-acting and long-acting GLP-1 receptor agonists can reduce the levels of FPG, 2hPG, and HbAlc, which is consistent with previous studies (31). Kapodistria’s study (32) showed that Liraglutide could promote enterocytes to secrete insulin by elevating endogenous GLP-1 levels from a physiological dose to a pharmacological dose. Actually, long-acting GLP-1 receptor agonists can provide better blood glucose control than short-acting ones because patients with long-acting receptor agonists have higher fasting insulin levels (possibly at night) (33, 34). Persistent high plasma levels of long-acting GLP-1 receptor agonists lead to a decrease in plasma HbA1c levels, which is greater than the decrease observed in intermittent activation of GLP-1 receptor caused by the administration of short-acting compounds (13, 35). Moreover, long-acting GLP-1 receptor agonists have no substantial effect on gastric motility, 76 which may be due to rapid immune response, which means that the effect of these compounds on gastric emptying decreases rapidly over time because they continuously activate GLP-1 receptor (36). In addition, long-acting GLP-1 receptor agonists do not reduce postprandial blood glucose fluctuations like short-acting compounds (37). by comparison, the clinical application of Liraglutide requires patients to inject the solution once a day. The drug is expensive with relatively low-cost performance, which limits its clinical use and makes it difficult to promote its application in primary hospitals.

In response to the expensive price and relatively low-cost performance of Liraglutide, Dulaglutide has begun to be used clinically (38). In our study, the levels of FPG, 2hPG, and HbAlc were decreased in both groups after the treatment for the period of 24 weeks. There was no statistical difference in blood glucose levels between the observation group and the control group after the treatment with Dulaglutide for the period of 24 weeks. However, the levels of visceral adiponectin, LP, FINS, and HOMA-IR were lower in both groups after treatment for 24 weeks than before the treatment. Cardiovascular disease caused by diabetes is one of the common complications of T2DM. Lipoprotein rich in cholesterol is an important risk factor for atherosclerosis, including coronary heart disease, myocardial infarction, stroke and peripheral vascular disease. Low density lipoprotein (LDL) and lipoprotein (a) Lp (a) are important components of cholesterol ester rich lipoproteins (39, 40). Kotani et al. found that endothelial dysfunction may be related to oxidized Lp (a) in T2DM patients (41). Saeed et al. studied the relationship between elevated Lp (a) and CVD risk in nearly 10000 male and female participants, including 1543 people with diabetes or pre diabetes (42). No statistical difference was determined in biochemical indices and HOMA-IR index levels between the observation group and the control group after the treatment for the period of 24 weeks. Dulaglutide can be applied to control blood glucose in type 2 diabetic patients. Because of the relatively high molecular weight of the injection solution, it is generally not easily absorbed and degraded by the body, thus the duration of drug activity is relatively long. Therefore, Dulaglutide can be used once a week to meet the clinical requirements. In addition, the solution can promote the release of insulin, delay gastric emptying, and control the total daily energy intake in a certain range by reducing the intake of food, to achieve a good hypoglycemic effect (43).

Pharmacoeconomics is the specific application of economic principles and methods in pharmaceuticals (44). By a broad generalized definition, pharmacoeconomics focuses on the study of the economic behavior of the supply and demand of drugs, the interaction between supply and demand of drug market pricing, and the measures of various intervention policies in the field of drugs (45). In a narrow sense, however, pharmacoeconomics is the application of the basic principles, methods, and analytical techniques of economics in the clinical treatment process of the drug, using the pharmacoepidemiological population as a guide and based on a society-wide perspective to seek maximum rational utilization (46). To analyze further the pharmacoeconomic value of Dulaglutide, we evaluated its use in this study from the perspective of cost and CER. We found no statistical difference between the two groups in terms of CER levels; the cost of the observation group was lower than that of the control group after the treatment for the period of 24 weeks.

In previous studies, it was found that the economic benefit of dulaglutide is higher than that of liraglutide in the short term, but the long-term economic benefit is still unclear. Our research results extend the previous results (12).Moreover, the economic value of Dulaglutide was higher, and the drug was relatively cost-effective compared with Liraglutide. Some researchers (47) gave Dulaglutide and Liraglutide to patients with type 2 diabetes and then evaluated the effect from the perspective of economics. Our research conclusion is consistent with the previous study. Their results also found that Dulaglutide has a price advantage for type 2 diabetic patients because Dulaglutide has a relatively low cost coupled with the fact that the solution is injected once a week and, therefore, is suitable for promotion in primary hospitals. In this study, the incidence of nausea and vomiting, hypoglycemia, cholecystitis, allergic reactions, and hepatic and renal abnormalities was lower in the observation group than in the control group during the treatment period, the complications of diabetes and its high hospitalization rate are important factors for the increase in treatment costs of diabetes (48). The gastrointestinal reaction of dulaglutide is significantly reduced. The weekly injection rate can improve the compliance of patients, reduce the incidence of complications of diabetes (20), and reduce the cost of consumables such as injection needles and diabetes management (48).

Therefore, patients with type 2 diabetes should be treated to improve relevant glucose and insulin indices then, appropriate hypoglycemic drugs should be selected in combination with their economic status and family background, to improve the pertinent treatment.

## 5 Conclusions

Both Liraglutide and Dulaglutide can reduce blood glucose level and improve visceral adiponectin, Lp, and the HOMA-IR index level in type 2 diabetic patients. The effects of Liraglutide and Dulaglutide are similar. Because Dulaglutide is more cost-effective and safer with fewer adverse reactions, the application of Dulaglutide deserves further promotion.

## Data availability statement

The original contributions presented in the study are included in the article/supplementary material. Further inquiries can be directed to the corresponding author.

## Ethics statement

This study was approved by the Ethics Committee of Ethics Committee of Longhu Hospital, First Affiliated Hospital of Medical College of Shantou University (the registration number is: ChiCTR1900026514). The patients/participants provided their written informed consent to participate in this study.

## Author contributions

Conceptualization, KH and YS; methodology, KH; formal analysis, FWC and JC; investigation, ZZW and FYD; resources, DZ; data curation, SZ; writing-original draft preparation, SZ and KH; writing-review and editing, YS, ZZW, WL; supervision, WL, FWC; funding acquisition, KH; All authors contributed to the article and approved the submitted version.
